# Combined PD-1/PD-L1 and tumor-infiltrating immune cells redefined a unique molecular subtype of high-grade serous ovarian carcinoma

**DOI:** 10.1186/s12864-021-08265-y

**Published:** 2022-01-13

**Authors:** Ping Liu, Ruoxu Chen, Xudong Zhang, Ruiting Fu, Lin Tao, Wei Jia

**Affiliations:** 1 First Affiliated Hospital, School of Medicine, Shihezi University/Department of Pathology and Key Laboratory for Xinjiang Endemic and Ethnic Diseases, Shihezi University School of Medicine, Shihezi, China; 2 Department of Obstetrics and Gynecology, First Affiliated Hospital, School of Medicine, Shihezi University, Shihezi, China

**Keywords:** Antigen-presenting cells, High-grade serous ovarian carcinoma, Immune cell infiltration, PD-1, PD-L1

## Abstract

**Background:**

High-grade serous ovarian carcinoma is highly heterogeneous, and although many studies have been conducted to identify high-grade serous ovarian carcinoma molecular subtypes that are sensitive to immunotherapy, no precise molecular subtype has been proposed to date. Immune cell infiltration and immune checkpoints are highly correlated with immunotherapy. Here, we investigated immune cell infiltration and immune checkpoint values for prognosis and precise immunotherapy for high-grade serous ovarian carcinoma based on molecular subtype classification.

**Results:**

“High antigen-presenting cells infiltration molecular subtype of high-grade serous ovarian carcinoma” was identified in immune cell infiltration profiles. Each of the three immune cell infiltration clusters (A, B, and C) demonstrated distinct immune cell characterization, with immune cell infiltration cluster C exhibiting high antigen-presenting cell infiltration, improved prognosis, and higher sensitivity to immunotherapy. Programmed death-1/programmed death ligand 1 has a prognostic and predictive role that can help classify molecular subtypes.

**Conclusions:**

Our findings redefined a unique molecular subtype of high-grade serous ovarian carcinoma, suggesting that high-grade serous ovarian carcinoma patients with higher antigen-presenting cell infiltration and programmed death-1/programmed death ligand 1 expression can benefit from precise immunotherapy.

**Supplementary Information:**

The online version contains supplementary material available at 10.1186/s12864-021-08265-y.

## Background

Epithelial ovarian carcinoma (EOC) remains the dominant cause of death among gynecological malignancies [1]. High-grade serous ovarian carcinoma (HGSOC) is mainly diagnosed at advanced stages (III/IV) with a 5-year survival rate of 45.6% and accounts for 90% of EOC cases [2, 3]. High heterogeneity and resistance to platinum chemotherapy significantly contribute to the poor prognosis of HGSOC [4]. Therefore, new molecular targets for the precise treatment of HGSOC need to be explored.

Recently, precise molecular treatment has proved effective and safe against gynecological malignant tumors, such as breast ductal carcinoma and endometrial carcinoma. In particular, the precise molecular classification of tumors contributed to treatment benefits, with the 5-year disease-free survival of breast ductal carcinoma luminal A and B subtypes being significantly higher than that of triple-negative and Her-2 positive subtypes [5, 6]. A global consensus has been reached regarding the treatment of endometrial carcinoma, stating that the POLE-ultramutated subtype of endometrial carcinoma with 98% 5-year recurrence-free survival and no need for adjuvant treatment had a better prognosis than other molecular subtypes [7, 8].

Meanwhile, numerous studies have focused on exploring the substantial molecular subtypes of HGSOC. Based on the tumor microenvironment (TME) or the infiltration of carcinoma-associated fibroblasts (CAFs) with distinct prognoses, HGSOC was categorized into three distinct subtypes: non-immune group, CAF-immune subtype, and non-CAF-immune subtype [9]. By analyzing the mRNA transcriptome, HGSOC can be divided into four transcriptomic subtypes: differentiated, immunoreactive, mesenchymal, and proliferative [10]. Additionally, by integrating transcriptomics and proteomics, HGSOC can be classified into two subtypes: high sensitivity to platinum therapy and low sensitivity to platinum therapy [11]. However, regardless of the immune signatures, transcriptome, or proteomics in the TME, the robustness, availability, and variability of these subtypes remain controversial, and it is difficult to reach a consensus on the targeted treatment for the molecular subtypes of HGSOC. Therefore, identifying the potential molecular subtypes indicative of HGSOC burden and therapies is essential.

Programmed death-1 (PD-1, CD279, PDCD1) is an immune checkpoint receptor of the CD28 family and is expressed on the surface of activated T cells, B cells, and other immune cells, especially dendritic cells (DCs). PD-1 is a receptor for programmed death ligand 1 (PD-L1, CD274, B7-H1), which is expressed on the surface of tumor cells [12]. Anti-PD-1 and its ligand PD-L1 have become critical to immunotherapy strategies in advanced malignant tumors, including melanoma, non-small cell lung carcinoma, urothelial carcinoma, renal cell carcinoma, and head and neck carcinoma, and are associated with improved outcome and survival [13–17]. The interaction of PD-1 and PD-L1 can prevent T cell activation by downregulating the function of tumor-infiltrating immune cells (TIICs) and weakening tumor immunogenicity [18]. In a previous study, PD-L1 expression was positive in 34.9% of all EOC subtypes and 57.4% of HGSOC subtypes [19]. In addition, PD-L1 was poorly expressed in tissue biopsies but was more prevalent in tumor-infiltrating cells than in tumor cells in HGSOC tissue sections [20, 21]. Patients with PD-L1 expression may be more sensitive to immunotherapy. Therefore, tumor immunogenicity and PD-1/PD-L1 expression are essential features for the molecular classification of HGSOC.

Notably, tumor immunogenicity can be weakened by the interaction of PD-1 and PD-L1 by impairing the functions of immune cells. However, APCs, which are vital components of TIICs, can improve immunogenicity by activating naïve T cells. This suggests that patients with a “high immunogenicity molecular subtype of HGSOC” might benefit from immunotherapy. To this end, we combined PD-1/PD-L1 with TIICs to explore the potential molecular subtypes of HGSOC.

## Results

### PD-1 and PD-L1 were associated with improved outcomes in HGSOC

**Fig. 1:**
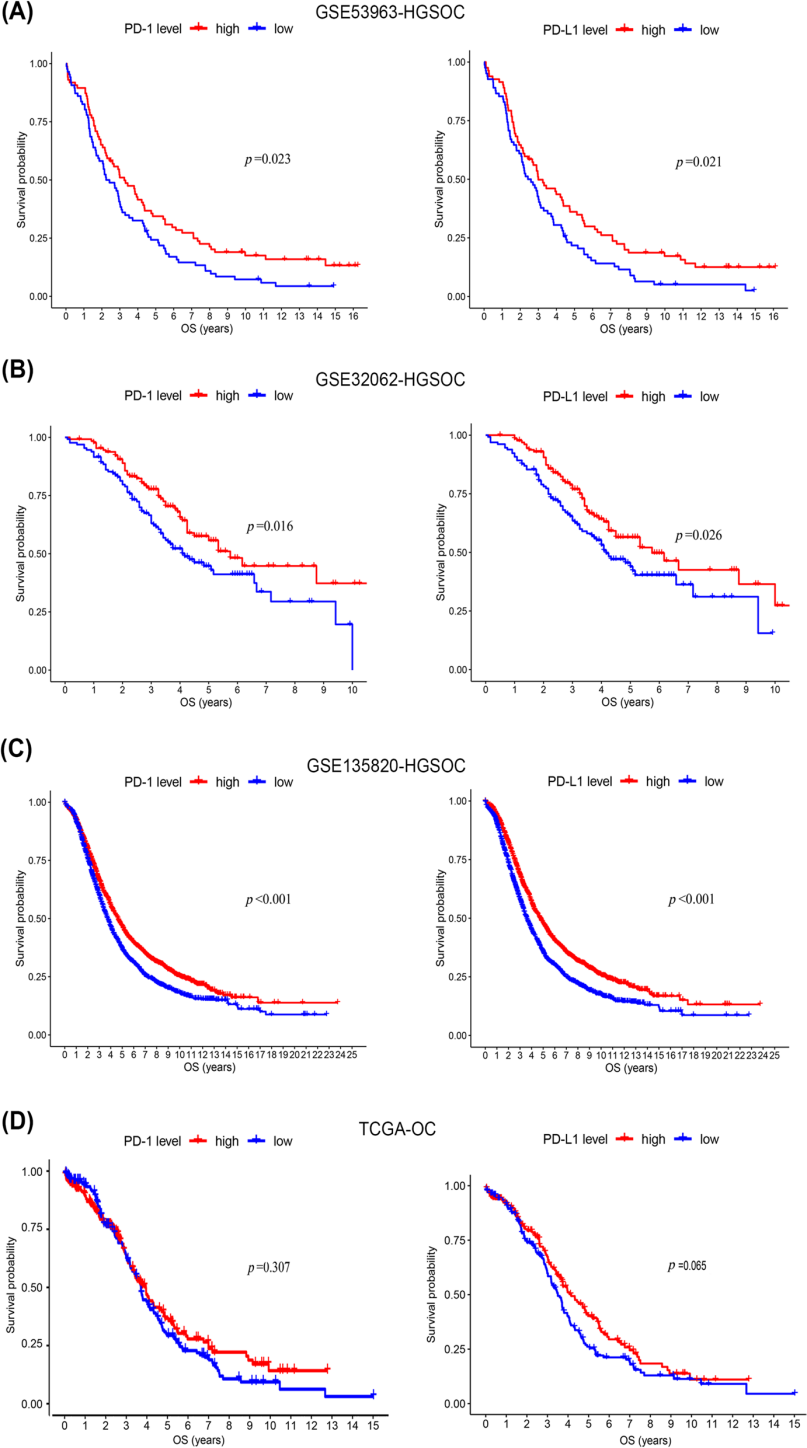
Prognostic significance of PD-1/PD-L1 in HGSOC. Kaplan-Meier analysis for overall survival of patients with higher (red) or lower (blue) PD-1/PD-L1 expression in the GSE53963 (**A**), GSE32062 (**B**), GSE135820 (**C**), and TCGA (D) databases. The log-rank test was used to compare groups, and *p* < 0.05 was considered significant. *HGSOC, High-grade serous ovarian carcinoma; PD-1, programmed death-1; PD-L1, programmed death ligand 1; TCGA, The Cancer General Atlas*

To investigate whether PD-1/PD-L1 expression was related to clinical prognosis, clinical information was extracted from four databases, including three HGSOC databases: GSE53963 (GSE53963-HGSOC), GSE135820 (GSE135820-HGSOC), and GSE32062 (GSE32062-HGSOC) and The Cancer General Atlas (TCGA) database of OC (TCGA-OC). Based on PD-1/PD-L1 expression levels from each database, the average score was set as a cutoff value dividing patients into two groups: higher and lower expression of PD-1/PD-L1. The higher PD-1/PD-L1 expression group had significantly better overall survival (OS) in the three HGSOC databases compared with the lower expression group (*p* = 0.023 for PD-1 and *p* = 0.021 for PD-L1 in GSE5396, *p* = 0.016 for PD-1 and *p* = 0.026 for PD-L1 in GSE32062, *p* < 0.001 for PD-1 and *p* < 0.001 for PD-L1 in GSE135820, log-rank test; Fig. 1A–C). No statistically significant difference in prognosis was obtained between the higher and the lower PD-1/PD-L1 expression groups of TCGA-OV (*p* = 0.307 for PD-1, *p* = 0.065 for PD-L1, log-rank test; Fig. 1D).

### Differentially expressed PD-1/PD-L1-related genes in HGSOC

**Fig. 2:**
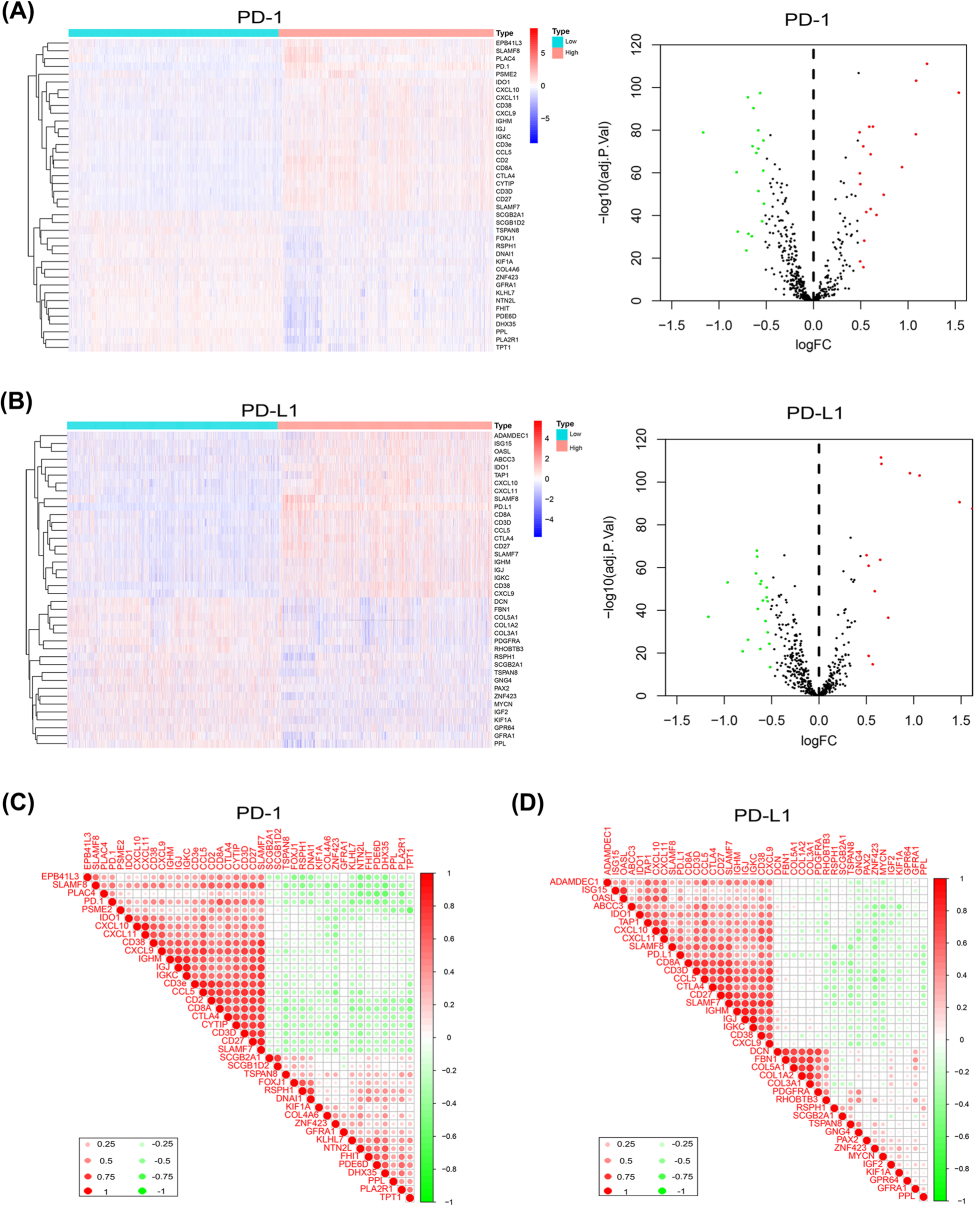
DEGs were compared between relatively higher expression and lower expression groups of PD-1/PD-L1 by analyzing the microarray database GSE135820. Heatmaps show DEGs that were related to PD-1 (**A**) or PD-L1 (**B**) in the two groups. Red indicates DEGs that were positively associated with PD-1/PD-L1, white indicates DEGs that were not related to PD-1/P-L1, and blue indicates DEGs that were negatively related to PD-1/P-L1. Volcano plot demonstrates different scattering of genes between high and low expression cluster groups of PD-1 or PD-L1. Genes (depicted in green or red) showed a fold change difference of > 0.5 and *p* < 0.05. Correlations of DEGs that were related to PD-1 (**C**) or PD-L1 (**D**) between two groups are shown in the correlation heatmap. Red indicates a positive correlation (*r* = 1), white indicates no correlation (*r* = 0), and green indicates a negative correlation (*r* = −1). *DEGs, differentially expressed genes; PD-1, programmed death-1; PD-L1, programmed death ligand 1*

To identify DEGs that were relevant to PD-1/PD-L1 expression, PD-1/PD-L1 was identified and screened in the GSE135820 microarray database after normalizing the chip results. Based on the PD-1/PD-L1 expression level, every sample from the microarray database was divided into two groups: higher and lower PD-1/PD-L1 expression groups. Keeping the cutoff value strict, genes with |logFC| > 0.5 of their expression between the two groups were considered DEGs and were depicted in a volcano plot. The top 20 DEGs that were conspicuously related to PD-1/PD-L1 are illustrated in a heatmap (Fig. 2A, B). Most of these DEGs were associated with the encoding of antigens on lymphatic cell membranes. The “Corrplot” R package was used to further investigate the correlations of DEGs. PD-1 was positively related to CD8A, CTLA4, and CD3D mRNA expression, and PD-L1 was significantly related to CTLA4, CD38, and CXCL9 mRNA expression (Fig. 2C, D).

### DEGs were closely associated with lymphocyte differentiation

**Fig. 3:**
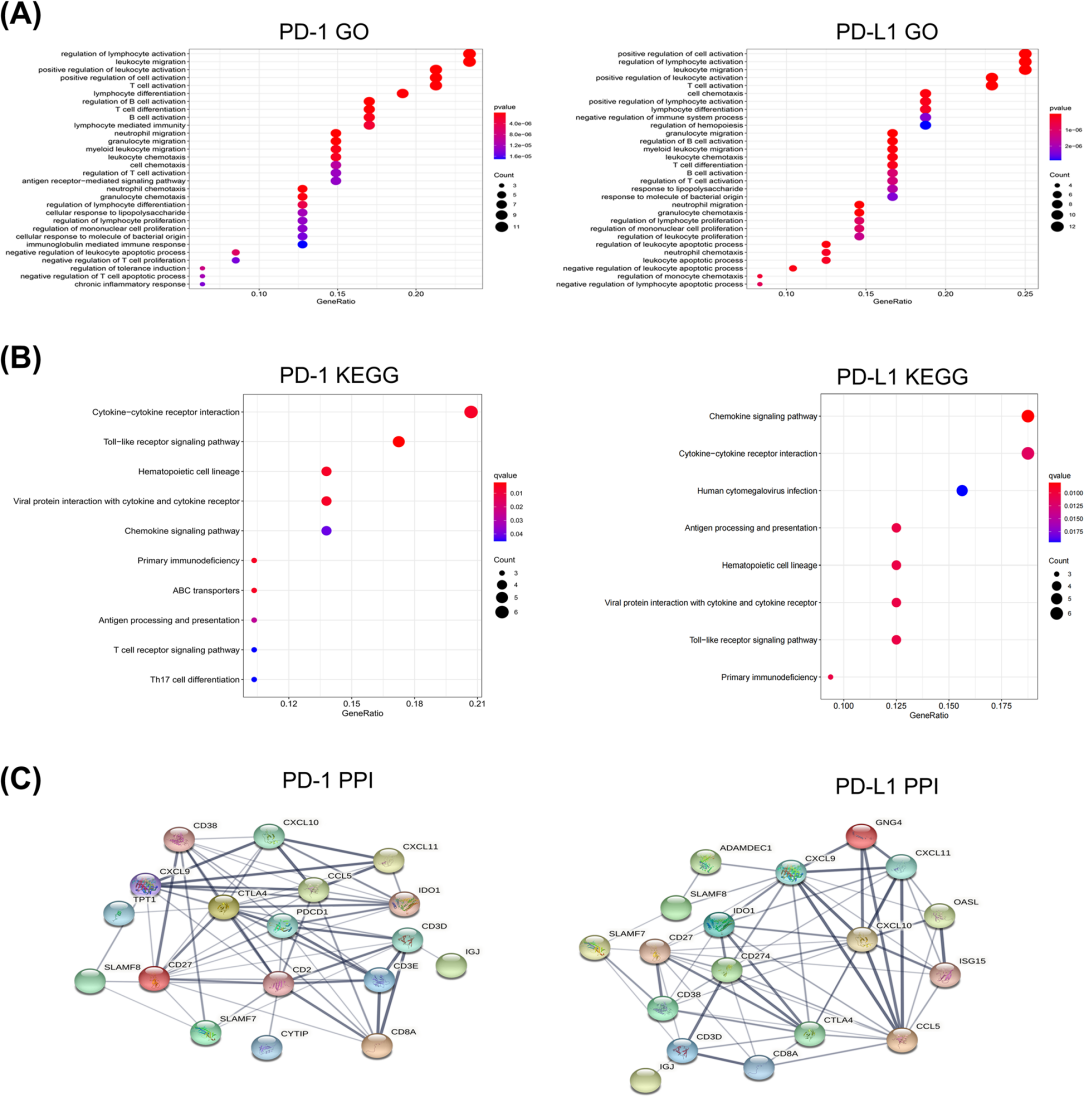
Functional analysis of DEGs associated with PD-1/PD-L1. **A** GO analysis of DEGs. **B** KEGG pathway analysis of DEGs. **C** PPI analysis of DEGs. The plot sizes indicate gene counts enriched in the pathway and the color depth shows the *p*-value from low (red) to high (blue) level. Significance was set at *p* < 0.05. *DEGs, differentially expressed genes; GO, Gene Ontology; KEGG, Kyoto Encyclopedia of Genes and Genomes; PPI, protein–protein interaction; PD-1, programmed death-1; PD-L1, programmed death ligand 1*

Functional enrichment analysis of DEGs was performed to identify a heterogenic intrinsic trait of HGSOC. PD-1/PD-L1 related DEG function analysis was performed using GO, KEGG, and PPI online tools (Fig. 3). In GO analysis, lymphocyte-associated pathways, including T cell and B cell, were the most enriched in both independent experimental groups of PD-1 and PD-L1 (Fig. 3A). In KEGG analysis, DEGs were mainly enriched in proinflammatory chemokine and cytokine-associated pathways, including chemokine signaling pathway, cytokine-cytokine receptor interaction, and toll-like receptor interaction (Fig. 3B). The online database “STRING” was further used to create a visible network to elucidate the structural and functional signatures of proteins (Fig. 3C). All pathways generated by KEGG, GO, and PPI analyses demonstrated lymphocyte differentiation.

### Higher APCs infiltrating fraction in HGSOC

**Fig. 4:**
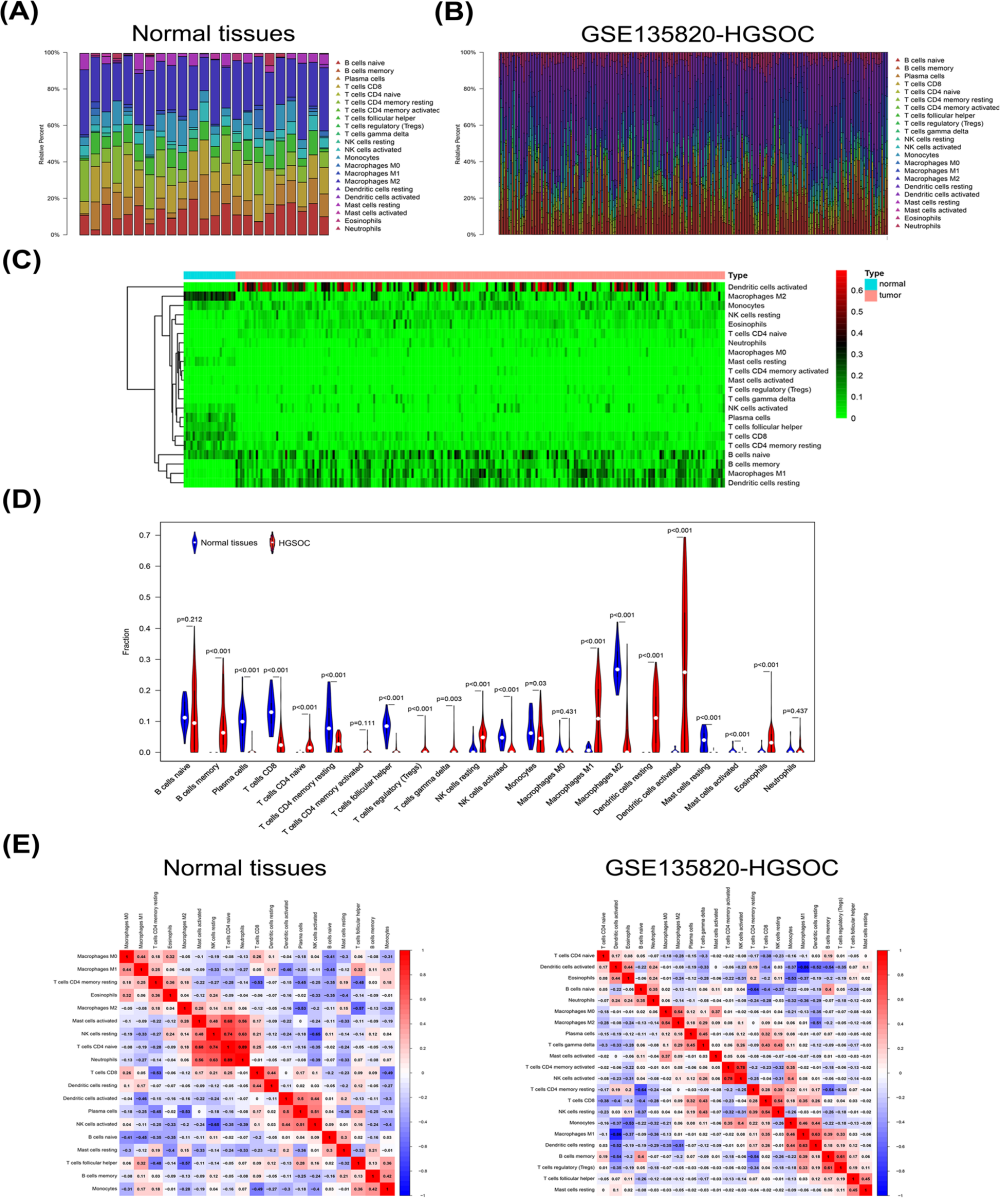
Assessment of the proportions of LM22 infiltrating immune cells. **A**, **B** Bar plots exhibit the immune cell proportion differences in normal tissues and GSE135820-HGSOC. **C**, **D** Differences in LM22 immune cell fractions between HGSOC and normal tissues are demonstrated in the heat map and Violin plot. **E** Relevance of LM22 infiltrating immune cells in normal tissues and GSE135820-HGSOC are shown in the correlation matrix. *HGSOC, High-grade serous ovarian carcinoma; LM22, leukocyte gene signature matrix*

Samples with CIBERSORT *p* ≥ 0.05 were eliminated, and infiltrating proportions of 22 immune cells were depicted and calculated in each database (Table 1). The fractions of activated memory CD4 T cells, regulatory T cells, and delta gamma T cells were excluded from the subsequent analysis as they had zero proportion in normal tissue samples. The organization of LM22 infiltrating immune cells in 23 normal and 239 HGSOC samples is summarized in Fig. 4A and B. Compared to normal tissues, infiltrating immune cell patterns of HGSOC are depicted in a heatmap (Fig. 4C), and the majority of LM22 infiltrating immune cell fractions were predominant in HGSOC groups (Fig. 4D). The proportions of memory B cells, naïve CD4 T cells, regulatory T cells, gamma delta T cells, resting NK cells, M1 macrophages, activated DCs, resting DCs, and eosinophils were significantly increased in HGSOC, whereas plasma cells, resting memory CD4 cells, follicular helper T cells, activated NK cells, monocytes, M2 macrophages, resting mast cells, and activated mast cells decreased compared to normal tissues. Compared to other EOC histological subtypes, DCs, M1 macrophages, and B cells had a notably higher proportion in LM22 immune cells in HGSOC, whereas CD8 T cells showed the opposite trend (Table 1). Relationships among LM22 immune cells in normal tissues and HGSOC were investigated. For instance, M1 macrophages were highly positively associated with M0 macrophages and strongly negatively related to activated DCs in normal tissues. In the GSE135850-HGSOC database, M1 macrophages were highly positively related to resting DCs and negatively related to activated DCs in the immune phenotype profiles (Fig. 4E). Similar phenomena were found in GES32062-HGSOC, GSE53963-HGSOC, and serous OC databases (Fig. S1A, B). The relationship and patterns of immune infiltrating cells in other EOC histological types are shown in the supplemental materials (Fig. S1C–F). HGSOC has a unique immune infiltrating pattern, which is significantly different from that of other EOC subtypes.

**Table 1:** Proportion of immune infiltrating cells in the HGSOC (GSE135820, GSE53963, GSE32062), normal, serous, endometrioid, clear cell, and TCGA-OC databases. A difference in the proportions of immune-infiltrating cells was observed between HGSOC-135820 and the other databases. (*p < 0.05, **p < 0.01, ***p < 0.001)

Type	HGSOC			Normal	Serous	Endometrioid	Clear cell	TCGA-OC
	GSE135820	GSE53963	GSE32062					
B cells naive	11.27%	11.15%	12.52%	12.24%	1.43%***	6.05%**	1.21%***	4.98%***
B cells memory	8.27%	12.80%	7.02%*	0.01%***	4.96%*	3.90%***	7.87%	1.31%***
Plasma cells	0.22%	0.00%	0.39%	9.17%***	10.57%***	11.21%***	8.62%***	2.63%***
T cells CD8	3.35%	3.60%	3.19%	12.98%***	7.39%**	10.00%***	9.13%	8.75%***
T cells CD4 naive	2.04%	2.01%	1.55%	0.03%***	3.93%	0.95%***	0.78%***	0.00%***
T cells CD4 memory resting	2.79%	1.75%	2.71%*	8.93%***	2.81%	9.26%**	8.62%	17.73%***
T cells CD4 memory activated	0.32%	0.01%	0.31%	0.00%*	7.19%*	3.37%***	5.91%***	1.40%***
T cells follicular helper	0.38%	1.51%	0.32%**	8.11%***	4.93%***	7.63%***	4.03%*	3.44%***
T cells regulatory (Tregs)	0.91%	2.26%	1.00%	0.00%***	0.95%***	1.21%	2.4%*	3.42%***
T cells gamma delta	1.03%	0.43%	1.11%	0.00%**	2.16%	2.66%	3.23%	0.28%***
NK cells resting	4.85%	3.69%	4.60%	1.20%***	4.31%	1.01%***	3.96%	0.34%***
NK cells activated	1.44%	1.19%	1.26%	4.65%***	4.18%*	8.50%***	3.52%*	3.72%***
Monocytes	5.63%	4.04%	6.76%	7.49%*	2.11%***	3.27%**	8.44%	2.58%***
Macrophages M0	0.55%	0.28%	0.51%	0.91%***	6.90%***	5.48%***	7.19%***	19.30%***
Macrophages M1	11.18%	13.32%	13.21%	0.98%***	6.95%***	4.08%**	5.52%*	7.85%
Macrophages M2	4.63%	3.91%	5.91%	28.03%***	4.76%	2.97%	5.30%	15.76%***
Dendritic cells resting	11.20%	13.62%	14.41%	0.02%***	6.06%	2.43%***	1.30%***	0.74%***
Dendritic cells activated	25.22%	20.00%	17.70%	0.33%***	1.58%***	4.08%***	0.39%***	2.63%***
Mast cells resting	0.01%	0.00%	0.00%	3.57%***	5.44%***	6.48%***	5.11%***	1.38%***
Mast cells activated	0.01%	0.00%	0.00%	0.44%***	8.78%***	2.25%***	4.03%***	1.16%***
Eosinophils	3.58%	3.86%	3.74%	0.36%***	0.89%***	2.14%*	2.34%	0.08%***
Neutrophils	1.13%	0.55%	1.78%	0.57%	1.73%***	1.06%	1.11%	0.52%

### A potent immunogenicity ICI subtype of HGSOC was recognized

**Fig. 5:**
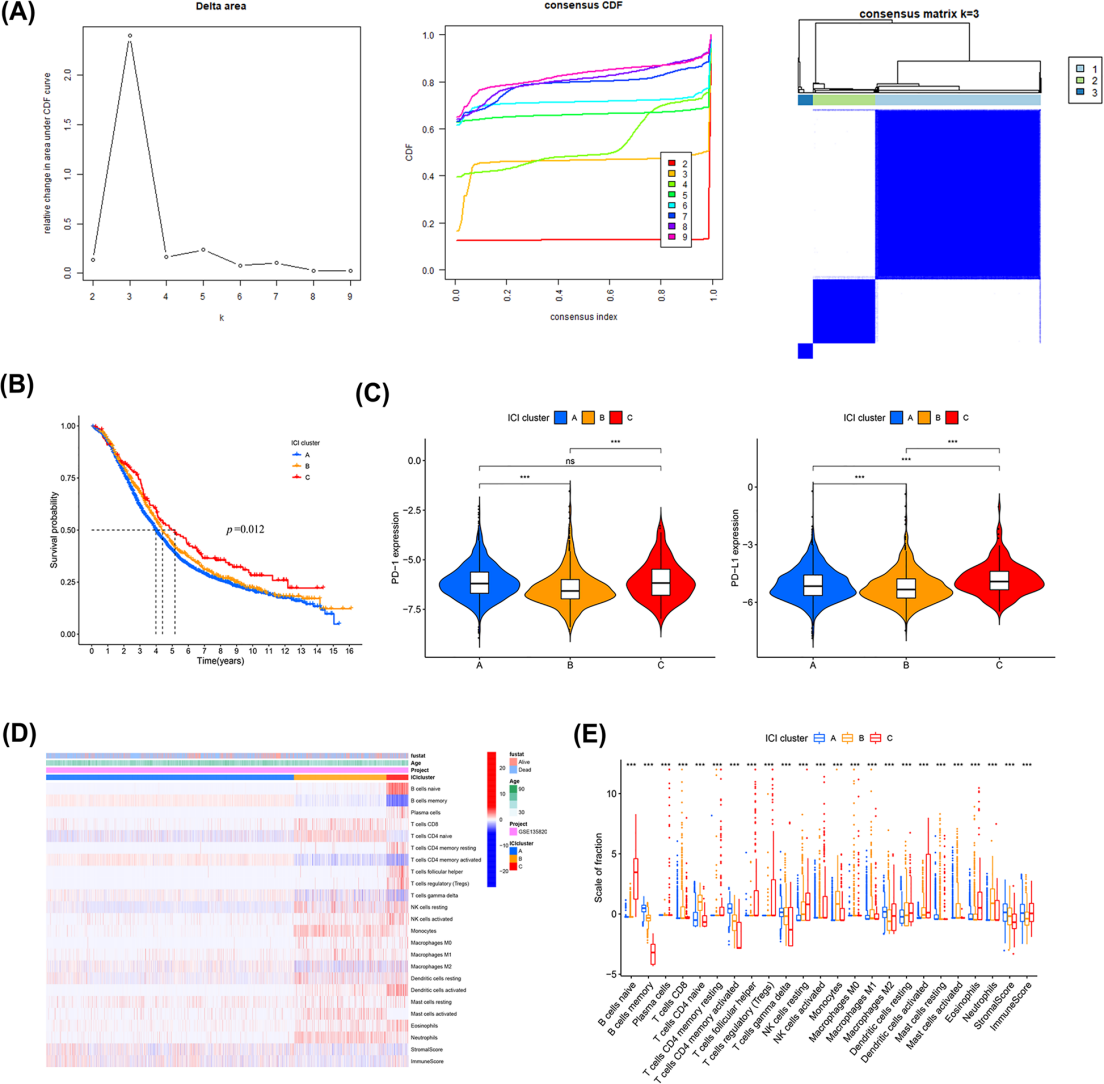
Differential clinicopathological features and survival of HGSOC in cluster A/B/C subtypes in GSE135820-HGSOC. **A** Consensus clustering matrix for k = 3. **B** Kaplan-Meier analysis of OS for patients with HGSOC in three clusters. **C** Differences in PD-1/PD-L1expression among three ICI clusters. **D** Unsupervised clustering of TIICs in GSE135820-HGSOC. Rows represent TIICs, and columns represent samples. **E** The fraction of TIICs in three ICI clusters. (Kruskal-Wallis test, *p < 0.05, **p < 0.01, ***p < 0.001, and ns *p* > 0.05). *HGSOC, High-grade serous ovarian carcinoma; ICI, immune cell infiltration; OS, overall survival; TIICs, tumor-infiltrating immune cells; PD-1, programmed death-1; PD-L1, programmed death ligand 1*

Based on CDF and delta curve analysis, immune cells could be classified into three optimal cluster groups (Fig. 5A). Meanwhile, 2873 HGSOC samples with CIBERSORT *p* < 0.05 were clustered into three subtypes: cluster 1/A (*n* = 1961), cluster 2/B (*n* = 737), and cluster 3/C (*n* = 175). In particular, cluster 3/C was a distinct portion (Table S4), with better outcomes, whereas ICI clusters A and B had poor outcomes (*p* = 0.012, log-rank test, Fig. 5B). Combined with the analysis of two immune checkpoints, PD-1 and PD-L1, in each group, ICI cluster C had a notably higher PD-1/PD-L1 expression level than ICI clusters A and B (*p* < 0.001, Kruskal-Wallis test, Fig. 5C). The distribution of immune cells in different cluster types is shown in the heatmap (Fig. 5D). Subsets of LM22 TIICs showed significant differences among the three cluster types (Fig. 5E); ICI cluster C was notable for a significantly higher proportion of naïve B cells, plasma cells, resting memory CD4 memory T cells, follicular helper T cells, regulatory T cells, resting NK cells, M1 macrophages, activated NK cells, activated DCs, and eosinophil infiltration. Cluster B was characterized by high levels of CD8 T cells, naïve CD4 T cells, monocytes, M1 macrophages, activated mast cells, and neutrophils. Cluster A had high levels of memory B cells, activated memory CD4 T cells, gamma delta T cells, and M2 macrophages. We concluded that ICI cluster C HGSOC patients with improved prognosis had higher APC infiltration and PD-1/PD-L1 expression levels.

### Combined PD-1/PD-L1 with DCs identified a potent immunogenic molecular subtype of HGSOC

**Fig. 6:**
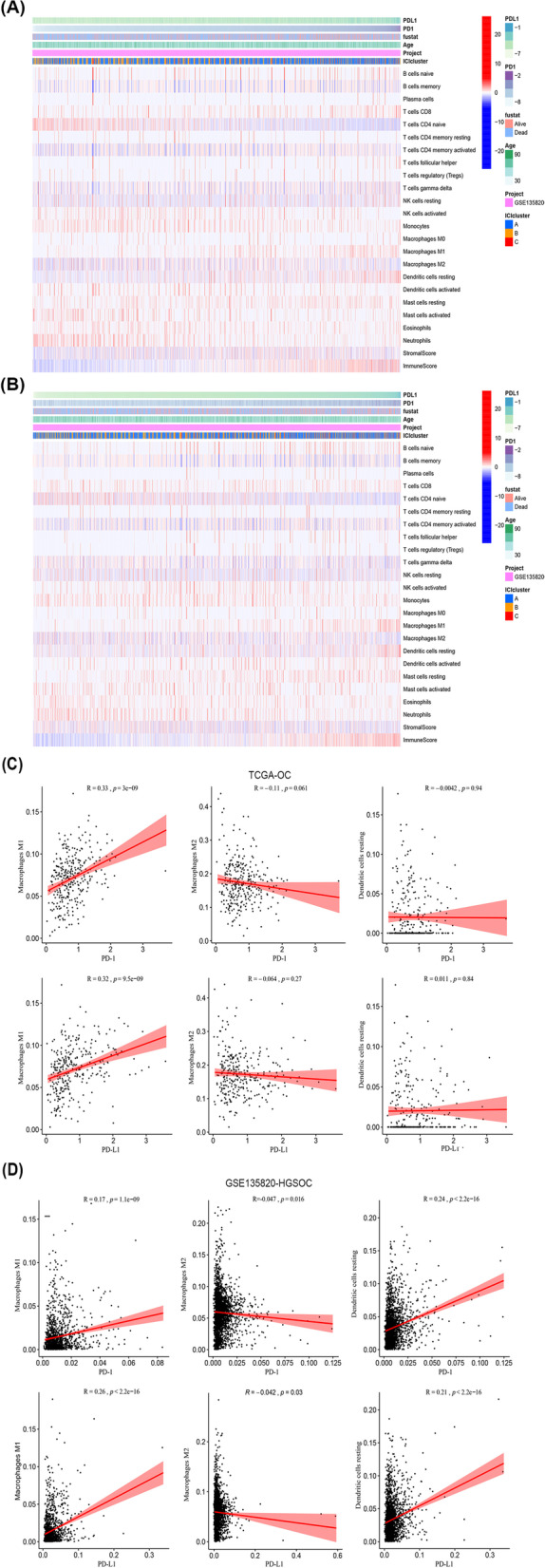
Correlation between PD-1/PD-L1 expression level and immune cell subsets. Heatmap indicates the correlation of PD-1 (**A**) and PD-L1 (**B**) with 22 immune cell subpopulations in GSE135820-HGSOC. Pearson coefficient correlation revealed the correlations between PD-1/PD-L1 expression levels and three immune cell subsets in TCGA-OC (**C**) and GSE135820-HGSOC (**D**). *HGSOC, High-grade serous ovarian carcinoma; OC, ovarian carcinoma; PD-1, programmed death-1; PD-L1, programmed death ligand 1*

To investigate infiltrating immune cells that could be associated with PD-1/PD-L1 expression level in HGSOC, the relationship between PD-1/PD-L1 and immune components in the TME was visualized (Fig. 6). Immune scores and several degrees of ICI (including mainly M1 macrophages, M2 macrophages, and resting DCs) seemed relevant to PD-1/PD-L1 expression levels (Fig. 6A, B). The relationship between the three immune cells and PD-1/PD-L1 was further investigated by Pearson correlation analysis. PD-1/PD-L1 expression was positively correlated to M1 macrophages in both TCGA-OC and GSE135820-HGSOC. Moreover, PD-1/PD-L1 was positively associated with resting DCs and negatively associated with M2 macrophages in GSE135820-HGSOC (Fig. 6D). However, no solid conclusion was made regarding the correlation of PD-1/PD-L1 with resting DCs and M2 macrophages in TCGA-OC (Fig. 6C). Similar results were found in the GSE53963 database of HGSOC (Fig. S2). We constructed a three factor/two level orthogonal experimental model to recalculate the correlation between overall survival and APC infiltrating cells in 239 HGSOC patients using SPSS software (Table S5). The overall survival of 239 HGSOC patients was related to the number of M1 macrophages, dendritic cells, and B cells (*p* < 0.01). The overall survival of 239 HGSOC patients was not affected by the three lines of APC infiltrating cells (*p* > 0.05).

## Discussion

PD-L1 is highly expressed in several advanced carcinomas and is a favorable prognostic candidate [22–24]. Several studies have demonstrated that higher expression of PD-L1 indicates a favorable prognosis and restored immunogenicity [25, 26], whereas some reports showed that PD-L1 expression is not associated with OS [27, 28]. The lack of adequate clinical samples and distinct experimental approaches can explain the ambiguity of whether high PD-L1 expression in HGSOC correlates with improved prognosis. In our study, we analyzed clinical data and PD-1/PD-L1 expression in a total of 4475 samples and reported that PD-1 and PD-L1 were both associated with a better prognosis of HGSOC. Compared with previous studies, our results are reproducible and predictable as we analyzed both PD-1 and PD-L1 expression in association with HGSOC survival using data from three independent HGSOC databases (GSE53963, GSE32062, and GSE135820). The high expression of PD-1 and PD-L1 in HGSOC in our study suggests that PD-1/PD-L1 expression could have a prognostic value in HGSOC.

PD-1/PD-L1 expression in HGSOC varies across individuals. To further clarify the factors influencing PD-1/PD-L1 expression in different HGSOC patients, some DEGs, such as cytotoxic T-lymphocyte-associated antigen 4 (CTLA-4), CXCL9, and CCL5, underwent bioinformatics analysis in our study. CTLA-4 is a common immune checkpoint inhibitor that prevents T cell activation by inhibiting the interaction of T cells with APCs [29]. T cell infiltration in the TME requires the co-expression and interaction of CCL5 and CXCL9 [30], and high expression of CXCL9 and PD-L1 is associated with longer OS [31]. The expression of DEGs regulates the infiltration ability of immune cells. Our study also showed that DEGs are associated with immune and inflammatory responses, chemotaxis and activation of lymphocytes, and cell adhesion. Collectively, these DEGs may affect the immune process by altering PD-1/PD-L1 expression, and the underlying component of immune cells could be considered a potential factor affecting PD-1/PD-L1 expression in HGSOC.

Immune cells in the TME during tumor immune response are not well understood; thus, the online tools CIBERSORT and ESTIMATE were applied to demonstrate the infiltrating patterns of immune cells and assess the value of the immune/stromal score of the immune response in various carcinomas [32]. Patients with higher immune/stromal scores had poor disease-free survival and a higher pathological T stage in prostate carcinoma [33]. Immunological components included prognostically relevant biomarkers of immune responses in HGSOC patients, and PD-1^+^ TIM-3^+^ CD8^+^ T cells demonstrated exhausted function and were associated with poor prognosis of HGSOC [34]. Evidence of a single EOC histological subtype regarding immune infiltration patterns has been poorly reported. A previous study used LM22 to evaluate the feasibility of leukocyte deconvolution from bulk tumors, including 547 genes that distinguish 22 human hematopoietic cell phenotypes [35]. Hence, investigating the patterns of LM22 immune cells is needed to reveal immune cell infiltration characteristics in HGSOC and other histological subtypes of EOC. Lower infiltration of T cells and M2 macrophages could be found in the TME of HGSOC. In addition, in the present study, a particularly higher APC (DCs, B cells, and M1 macrophages) infiltrating pattern in HGSOC was demonstrated compared with other histological types of EOC. Mature DCs are associated with favorable immune infiltrating patterns and improved outcomes in HGSOC patients [36]. Therefore, we clarified that HGSOC with a high APC infiltration pattern might have favorable clinical outcomes based on variations in ICI proportion among different EOC subtypes.

The ICI cluster classification method has been applied to a broad range of molecular subtypes of carcinoma because of its general usefulness in immunotherapy [37, 38]. Triple-negative breast carcinoma (TNBC) was identified in three immune molecular subtypes (immunity high, immunity medium, and immunity low) by ICI cluster classification. Identifying TNBC subtypes has potential clinical significance in TNBC treatment [39]. A special molecular subtype of HGSOC in patients with a high APC infiltrating pattern, which may be more beneficial for immune therapy, was further analyzed. HGSOC was further divided into three distinct ICI clusters based on this classification. Immune cells demonstrated distinct expression fractions of three ICI cluster groups: ICI cluster C with the highest APC infiltration had the best survival prognosis, ICI cluster B with intermediate APC infiltration had medium survival prognosis, whereas ICI cluster A with the lowest APC infiltration had poor outcomes. Interestingly, ICI cluster C, with a higher proportion of DCs, as a unique immune infiltrating subtype that differs from other ICI cluster groups, was observed in our study. DCs, which are antigen-presenting cells, can enhance tumor immunogenicity by identifying, engulfing, processing, and presenting tumor-associated antigens to activate naïve T cells [40]. Potent immunogenicity can contribute to an effective response to immunotherapy [41, 42]. Therefore, HGSOC patients in ICI cluster C with the highest APC infiltration might be more sensitive to immunotherapy. These results demonstrate the rationality of APC infiltration as the basis for immune molecular subtype classification.

To further investigate the association of PD-1/PD-L1 expression with immune cells in HGSOC, three ICI clusters showed significantly distinct PD-1/PD-L1 expression. Resting DCs had a significant positive correlation with PD-1/PD-L1 expression in HGSOC. Considering that DCs can improve immunogenicity during antigen presentation, the degree of infiltration of DCs and the potency of immunogenicity can be indicated by PD-1/PD-L1 expression. In contrast, clinically relevant immunosuppression can be demonstrated by M2 macrophages in the TME [43]. In our study, PD-1/ PD-L1 expression had a strong positive correlation with M1 macrophages and a negative correlation with M2 macrophages. High M1 and low M2 macrophage infiltration of OC is associated with improved survival [44–46]. Hence, the intrinsic interaction between PD-1/PD-L1 and DCs could improve the prognosis of HGSOC patients. Our findings demonstrate that PD-1/PD-L1 could be a reference biomarker for molecular subtype classification of HGSOC.

## Conclusions

Patients with high APC infiltration may have potent immunogenicity and a satisfactory prognosis. Comprehensive assessment of LM22 ICI models of HGSOC might facilitate the exploration of new molecular targets for precise immunotherapy. We elucidated that the “high APC infiltration molecular subtype of HGSOC” has high immunogenicity and can greatly benefit from anti-PD-1/PD-L1 immunotherapy. In addition, PD-1/PD-L1 expression level can be considered a predictor of the infiltrating level of APCs. However, the correlation between the molecular subtype of HGSOC with high APC infiltration and the expression of PD-1, PD-L1, survival, and prognosis of HGSOC patients requires further validation in independent tissue samples.

## Methods

### Data source and data processing

The RNA-seq transcriptome data and the corresponding clinical data of OC were downloaded from TCGA (https://portal.gdc.carcinoma.gov/) and Gene Expression Omnibus (GEO) (https://www.ncbi.nlm.nih.gov/geo/) data portals. Gene expression data of 88 normal ovarian samples were downloaded from the Genotype-Tissue Expression (GTEx) (https://commonfund.nih.gov/GTEx/) data portal. Gene expression profiles of nine databases: GSE53963 (*n* = 174), GSE32060 (*n* = 260), GSE135820 (*n* = 4041), GSE17260 (*n* = 110), GSE73614 (*n* = 107), GSE73638 (*n* = 102), GSE68600 (*n* = 113), TCGA database of OC (*n* = 378), and GTEx accession (*n* = 88) were obtained. Based on different histological types of EOC in four independent databases (GSE17260, GSE73614, GSE73638, and GSE68600), three recombinational EOC histological database samples, namely serous (*n* = 33), endometrioid (*n* = 25), and clear cell OC (*n* = 21), were collected from the four EOC databases. The normalized data were uploaded by the authors and retrieved from the aforementioned three data portals. The “Limma” R package was used to identify differentially expressed genes (DEGs). Gene probe IDs for which an “-/NA” notation was not available were removed.

### Survival analysis

Clinical survival information was collected from the GSE53963, GSE32062, GSE135820, and TCGA accessions. The clinical endpoint was OS, and this clinical information was downloaded from each dataset (Table S1). The “Survival” R package was applied to evaluate the prognostic value of PD-1 and PD-L1.

### Enrichment and protein-protein network analysis

Relative DEG lists were uploaded using the online tools Database for Annotation Visualization and Integrated Discovery (DAVID, david.ncifcrf.gov/) and STRING (https://string-db.org/), and the results of Gene Ontology (GO), Kyoto Encyclopedia of Genes and Genomes (KEGG) pathway, and protein-protein interaction (PPI) analyses were obtained. Datasets with |log fold change | ≥0.2 and adjusted *p* < 0.05 were deemed meaningful enrichment pathways. The “Cluster Profiler” R package was used to combine gene annotation and gene expression analyses results [47]. The results are visualized in a circular plot.

### Molecular subtypes identification and estimated fractions of the TIICs

The immune and stromal scores of each sample were assessed using the ESTIMATE algorithm of the “estimate” R package [48]. A leukocyte gene signature matrix (LM22) was designed and validated to assess the feasibility of leukocyte deconvolution from bulk tumors. It contains 547 genes that distinguish 22 human hematopoietic cell phenotypes, including seven T cell types, naïve and memory B cells, plasma cells, NK cells, and myeloid subsets [35]. The fractions of 22 immune cells from each sample were obtained by calculating the relative subsets of RNA transcripts. This approach mainly depends on the LM22. TIIC levels in EOC tissues were identified using the “CIBERSORT” R package [49]. The total proportion of 22 immune cells was one for every sample. Only samples with a CIBERSORT *p* < 0.05 were considered significant and subjected to subsequent analysis. The results for TIIC proportions and histological subtypes of EOC after processing and screening in each database are shown in Table S2. The consensus cluster algorithm was applied using the “ConsensuClusterPlus” R package to determine the potential clusters of TIICs [47].

### Correlation between PD-1/PD-L1 and TIICs

The average proportions of LM22 immune cell subsets in each microarray dataset are shown in Table S3. PD-1/PD-L1 expression profiles in GSE135820, GSE53963, GSE32062, and TCGA-OC were obtained. The correlation between PD-1/PD-L1 and TIICs was evaluated using the R package.

### Statistical analyses

OS differences were calculated using the log-rank statistic in R. Pearson correlation analysis was performed to determine the relevant LM22 immune cells in the three groups. The Wilcoxon test was used to compare immune cell infiltration levels in primary HGSOC and normal ovarian tissues. All statistical analyses were performed using R Version 4.0.2 (R-project.org), and R packages were obtained from the Bioconductor project (www.bioconductor.org). Statistical significance was set at *p* < 0.05.

## Supplementary Information

**Additional file 1: Figure S1:** Proportions and relevance of LM22 immune cells in other databases: (A) GSE32062-HGSOC, (B) GSE53963-HGSOC, (C) TCGA-OC, (D) Serous, (E) Endometrioid, and (F) Clear cell database. *HGSOC, High-grade serous ovarian carcinoma; LM22, leukocyte gene signature matrix; TCGA, The Cancer General Atlas*

**Additional file 2: Figure S2:** Correlation between PD-1/PD-L1 expression and three immune cells infiltrating levels: M1 macrophages, M2 macrophages, and resting dendritic cells in (A) GSE53963-HGSOC, (B) GSE32062-HGSOC. *HGSOC, High-grade serous ovarian carcinoma; PD-1, programmed death-1; PD-L1, programmed death ligand 1*

**Additional file 3: Table S1:** Clinical data for sample id, overall survival time, survival state, and the average expression of PD-1/PD-L1 in GSE53963, GSE32062, GSE135820, and TCGA-OV databases. *PD-1, programmed death-1; PD-L1, programmed death ligand 1; TCGA, The Cancer General Atlas*

**Additional file 4: Table S2:** Proportions of infiltrating immune cells of each sample in GSE53963, GSE32062, GSE135820, GSE17260, GSE73614, GSE68600, GSE73638, TCGA-OC, and GETx databases. *GTEx, Genotype-Tissue Expression*; *OC, Ovarian cancer; TCGA, The Cancer General Atlas*

**Additional file 5: Table S3:** Average proportions of 22 immune cells subsets in GSE135820-HGSOC, GETx-Normal, Serous, Endometrioid, Clear cell, and TCGA-OC databases. *GTEx, Genotype-Tissue Expression*; *HGSOC, High-grade serous ovarian carcinoma; OC, Ovarian cancer; TCGA, The Cancer General Atlas*

**Additional file 6: Table S4:** Immune cells could be classified into three optimal cluster groups in 2873 HGSOC samples with CIBERSORT: cluster 1/A (*n* = 1961), cluster 2/B (*n* = 737), and cluster 3/C (*n* = 175). *HGSOC, High-grade serous ovarian carcinoma*

**Additional file 7: Table S5:** Correlation between overall survival of 239 HGSOC patients and the content of APC infiltrating cells (M1 macrophages, dendritic cells, and B cells).

## Data Availability

The datasets used and/or analyzed during the current study are available from the corresponding author on reasonable request.

## Abbreviations

APCs: Antigen-presenting cells
CAF: Carcinoma-associated fibroblast
CDF: Cumulative distribution functions
CTLA-4: Cytotoxic T-lymphocyte-associated antigen 4
DC: Dendritic cell
DEGs: Differentially expressed genes
EC: Endometrial carcinoma
EOC: epithelial ovarian carcinoma
GEO: Gene Expression Omnibus
GO: Gene Ontology
GTEx: Genotype-Tissue Expression
HGSOC: High-grade serous ovarian carcinoma
ICI: Immune cell infiltration
KEGG: Kyoto Encyclopedia of Genes and Genomes
LM22: Leukocyte gene signature matrix
OC: Ovarian carcinoma
OS: Overall survival
PD-1: Programmed death-1
PD-L1: Programmed death ligand 1
PPI: Protein-protein interaction
TCGA: The Cancer General Atlas
TIIC: Tumor-infiltrating immune cells
TME: Tumor microenvironment
TNBC: Triple-negative breast carcinoma
