# Supplementary figures and images for: Combined PD-1/PD-L1 and tumor-infiltrating immune cells redefined a unique molecular subtype of high-grade serous ovarian carcinoma
Source: BMC Genomics. 2022 Jan 13;23:51. doi: 10.1186/s12864-021-08265-y (PMC8759258; doi:10.1186/s12864-021-08265-y)

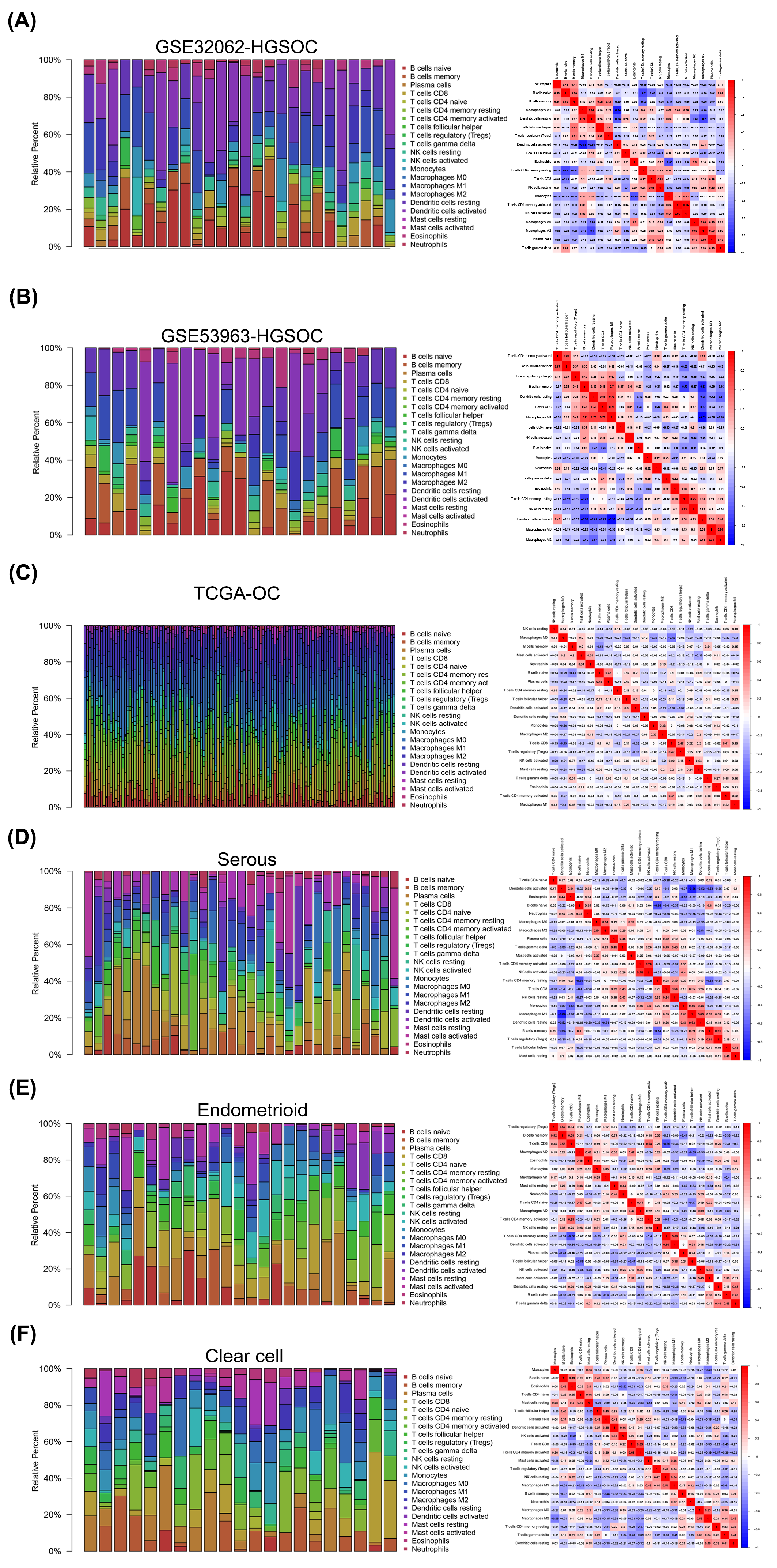

Supplement: Supplementary file 1 — Additional file 1: Figure S1: Proportions and relevance of LM22 immune cells in other databases: (A) GSE32062-HGSOC, (B) GSE53963-HGSOC, (C) TCGA-OC, (D) Serous, (E) Endometrioid, and (F) Clear cell database. HGSOC, High-grade serous ovarian carcinoma; LM22, leukocyte gene signature matrix; TCGA, The Cancer General Atlas [file 12864_2021_8265_MOESM1_ESM.tif]

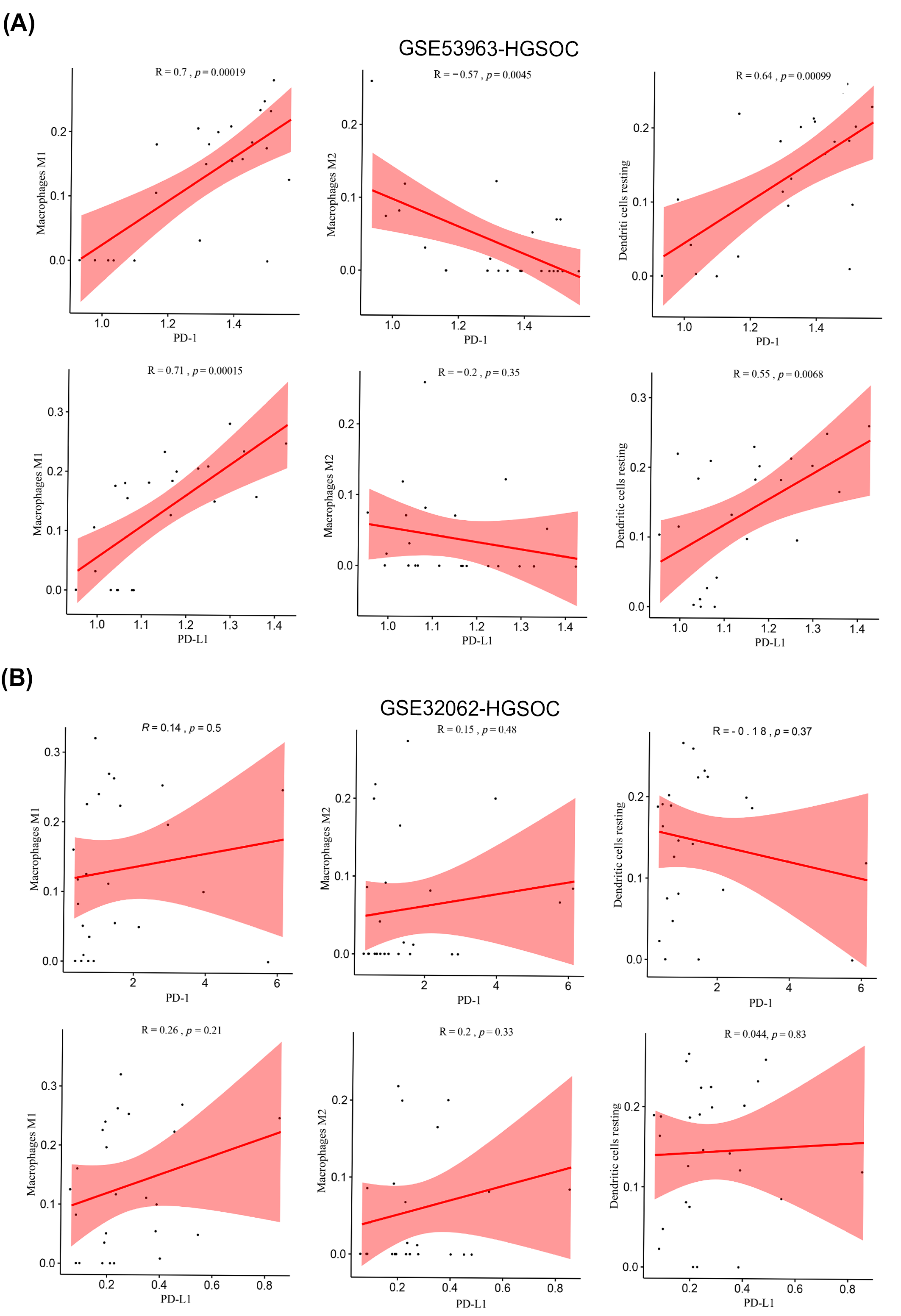

Supplement: Supplementary file 2 — Additional file 2: Figure S2: Correlation between PD-1/PD-L1 expression and three immune cells infiltrating levels: M1 macrophages, M2 macrophages, and resting dendritic cells in (A) GSE53963-HGSOC, (B) GSE32062-HGSOC. HGSOC, High-grade serous ovarian carcinoma; PD-1, programmed death-1; PD-L1, programmed death ligand 1 [file 12864_2021_8265_MOESM2_ESM.tif]
